# Classification of *Bartonella* Strains Associated with Straw-Colored Fruit Bats (*Eidolon helvum*) across Africa Using a Multi-locus Sequence Typing Platform

**DOI:** 10.1371/journal.pntd.0003478

**Published:** 2015-01-30

**Authors:** Ying Bai, David T. S. Hayman, Clifton D. McKee, Michael Y. Kosoy

**Affiliations:** 1 Division of Vector-Borne Diseases, Centers for Disease Control and Prevention, Fort Collins, Colorado, United States of America; 2 Molecular Epidemiology and Public Health Laboratory, Infectious Disease Research Centre, Massey University, Palmerston North, New Zealand; 3 Department of Biology, Colorado State University, Fort Collins, Colorado, United States of America; University of Texas Medical Branch, UNITED STATES

## Abstract

Bartonellae are facultative intracellular bacteria and are highly adapted to their mammalian host cell niches. Straw-colored fruit bats (*Eidolon helvum*) are commonly infected with several bartonella strains. To elucidate the genetic diversity of these bartonella strains, we analyzed 79 bartonella isolates from straw-colored fruit bats in seven countries across Africa (Cameroon, Annobon island of Equatorial Guinea, Ghana, Kenya, Nigeria, Tanzania, and Uganda) using a multi-locus sequencing typing (MLST) approach based on nucleotide sequences of eight loci (*ftsZ, gltA, nuoG, ribC, rpoB, ssrA*, ITS, and 16S rRNA). The analysis of each locus but ribC demonstrated clustering of the isolates into six genogroups (*E1 – E5* and *Ew*), while *ribC* was absent in the isolates belonging to the genogroup *Ew*. In general, grouping of all isolates by each locus was mutually supportive; however, *nuoG, gltA*, and *rpoB* showed some incongruity with other loci in several strains, suggesting a possibility of recombination events, which were confirmed by network analyses and recombination/mutation rate ratio (*r/m*) estimations. The MLST scheme revealed 45 unique sequence types (ST1 – 45) among the analyzed bartonella isolates. Phylogenetic analysis of concatenated sequences supported the discrimination of six phylogenetic lineages (*E1 – E5* and *Ew*) corresponding to separate and unique *Bartonella* species. One of the defined lineages, *Ew*, consisted of only two STs (ST1 and ST2), and comprised more than one-quarter of the analyzed isolates, while other lineages contained higher numbers of STs with a smaller number of isolates belonging to each lineage. The low number of allelic polymorphisms of isolates belonging to *Ew* suggests a more recent origin for this species. Our findings suggest that at least six *Bartonella* species are associated with straw-colored fruit bats, and that distinct STs can be found across the distribution of this bat species, including in populations of bats which are genetically distinct.

## Author Summary

Bats, with over 1000 recognized species, represent about 20% of all classified mammalian species worldwide. These mammals have a wide range of ecologies and life-history traits, and are now widely recognized as important reservoirs of many pathogens. *Bartonella* species have been found distributed in a wide range of mammalian species, including bats. About half of recognized *Bartonella* species, including one bat-associated species, have been associated with human illness. Previous studies have shown that *Bartonella* species are extremely diverse, with or without evident specificity to their mammalian hosts. Possessing many unique aspects, bartonellae can serve as a useful biological marker to study how microorganisms have evolved and diversified along with their animal hosts in evolutionary history. In this study, we applied multi-locus sequence typing, or MLST, to study the genetic differences of straw-colored fruit bat (*Eidolon helvum*)-associated *Bartonella* species. Our studies suggest *Bartonella* species have both exchanged genetic materials among species through recombination events and lost genes that are perhaps superfluous to their life cycles, which includes an intracellular stage in mammals.

## Introduction

Bartonellae are both Gram-negative alpha-proteobacteria and hemotropic bacteria highly adapted to facultative intracellular lifestyle in a wide variety of mammals, such as rodents, bats, insectivores, carnivores, ungulates, and other vertebrates. During the last two decades, progressively more bacterial species belonging to the genus *Bartonella* have been recognized with over 30 species described from different mammalian hosts. A number of *Bartonella* species were found to be associated with human illnesses and are associated with a growing spectrum of emerging diseases, including life-threatening endocarditis [1–9]. Animal reservoirs have been identified for some of the human pathogens, while remain unknown for others. Knowledge of the transmission of bartonella bacteria between mammalian hosts is incomplete. However, hematophagous arthropods such as fleas, flies, lice, mites, and ticks have been found naturally infected and are frequently implicated in transmitting *Bartonella* species [10–15].

Increasing recognition of bats as natural reservoirs of many emerging pathogens has drawn considerable attentions to study these mammals [16]. Multiple investigations of bartonella in bats have been conducted in different regions of the world [17–23]. These studies reported that bartonella infections are highly prevalent in many bat species and bartonella communities associated with bats are extremely diverse with co-circulation of numerous *Bartonella* species in the same bat populations. The data on relationships between *Bartonella* species and bats are quite contradictive depending on the investigated geographic region. Specifically, in Central and South America, multiple bat species may share the same *Bartonella* species without an evident host-specificity [19,20], while investigations from Asia and Africa demonstrated that a bat population typically harbors one or few *Bartonella* species specific for a particular bat species [17–18,21–22].

The straw-colored fruit bat (*Eidolon helvum*) is widely distributed in Africa. Colonies of these bats can be large and often found near human populations, with some roosts containing millions of individuals. Like other bats, straw-colored fruit bats have long life spans, with some individuals reaching at least 14 years of age [24]. Local human residents have a close contact with the bats, as they are frequently hunted for “bush meat”. A previous study in Kenya showed that straw-colored fruit bats are infected with bartonellae with high prevalence reaching 26% [18]. The same study demonstrated that *Bartonella* associated with the bats were genetically distant and belonged to four distinct genogroups based on sequence variation in the citrate synthase gene (*gltA*) [18]. Similar observations were reported in a very recent study conducted in Nigeria [22]. Considering the broad distribution and other ecological characteristics of straw-colored fruit bats, it was expected that additional *Bartonella* genogroups associated with this bat species can be identified in Africa.

In the present study, we aim to expand our knowledge of bartonella infections in straw-colored fruit bats and to better understand how multiple *Bartonella* species can co-habit populations of one bat species. We compared genetic differences of bartonella isolates obtained from straw-colored fruit bats captured in seven countries across Africa using a multi-locus sequence typing (MLST) approach. Based on comparison of nucleotide sequences derived from multiple loci, MLST has been shown to provide high discriminatory power in epidemiological and genetic analysis of bacterial strain populations, while retaining signatures of longer-term evolutionary relationships or clonal stability [25–28]. In addition, sequencing of multiple loci can detect evidences of micro-evolutionary events, particularly homologous recombination, among identified sequence types [29,30]. The main objective of the current study was to analyze bartonella isolates obtained from naturally infected straw-colored fruit bats from different parts of Africa to determine whether well-defined phylogenetic lineages correspond to currently accepted criteria for discrimination of bacterial species [31]. This, in turn, can help to enhance our understanding of population structure in the bacteria and the relationships between allelic profiles and the animal host. To achieve this goal, we developed a MLST scheme that incorporates eight loci which have been previously proposed for characterization of *Bartonella* species. In this study, clusters defined by the phylogenetic analysis were called either a “genogroup” or a “lineage”. A genogroup is defined when a single locus sequence was applied for the phylogenetic analysis, while a lineage is defined from concatenated sequences of all loci.

## Materials and Methods

### Bartonella isolates selection

A total of 79 isolates (Table 1) originating from straw-colored fruit bats from seven countries in different regions across Africa were selected for the MLST analysis, including Annobón island of Equatorial Guinea (n = 9), Cameroon (n = 3). Ghana (n = 20), Kenya (n = 16), Nigeria (n = 11), Tanzania (n = 15), and Uganda (n = 5). These isolates were obtained from previous studies completed by Bartonella Laboratory, CDC at Fort Collins, Colorado, and culturing procedures were published elsewhere [18,20].

**Table 1:** Allelic profiles, sequence types (ST), and lineage identification for the 79 *Bartonella* isolates from different geographic locations.

#	Isolate	Country/Region	ftsZ	gltA	nuoG	ribC	rpoB	ssrA	ITS	16S rRNA	Total length	ST	Lineage
1	B32133	Nigeria	1	1	1	neg	1	1	1	1	4622	ST1	Ew
2	B37799	Annobón	1	1	1	neg	1	1	1	1	4622	ST1	Ew
3	B37802	Annobón	1	1	1	neg	1	1	1	1	4622	ST1	Ew
4	B37890	Annobón	1	1	1	neg	1	1	1	1	4622	ST1	Ew
5	B37893	Annobón	1	1	1	neg	1	1	1	1	4622	ST1	Ew
6	B37895	Annobón	1	1	1	neg	1	1	1	1	4622	ST1	Ew
7	B37897	Annobón	1	1	1	neg	1	1	1	1	4622	ST1	Ew
8	B39231	Ghana	1	1	1	neg	1	1	1	1	4622	ST1	Ew
9	B39235	Ghana	1	1	1	neg	1	1	1	1	4622	ST1	Ew
10	B39243	Ghana	1	1	1	neg	1	1	1	1	4622	ST1	Ew
11	B39248	Ghana	1	1	1	neg	1	1	1	1	4622	ST1	Ew
12	B39957	Cameroon	1	1	1	neg	1	1	1	1	4622	ST1	Ew
13	B39958	Cameroon	1	1	1	neg	1	1	1	1	4622	ST1	Ew
14	B40389	Tanzania	1	1	1	neg	1	1	1	1	4622	ST1	Ew
15	B40404	Tanzania	1	1	1	neg	1	1	1	1	4622	ST1	Ew
16	B40906	Uganda	1	1	1	neg	1	1	1	1	4622	ST1	Ew
17	B40907	Uganda	1	1	1	neg	1	1	1	1	4622	ST1	Ew
18	B32135	Nigeria	1	1	2	neg	1	1	1	1	4622	ST2	Ew
19	B23978	Kenya	1	1	2	neg	1	1	1	1	4622	ST2	Ew
20	B39238	Ghana	1	1	2	neg	1	1	1	1	4622	ST2	Ew
21	B40386	Tanzania	1	1	2	neg	1	1	1	1	4622	ST2	Ew
22	B23975	Kenya	2	2	3	1*	2	4	2	2	5007	ST3	E5
23	B39233	Ghana	2	2	4	1*	2	5	4	2	5007	ST4	E5
24	B39296	Ghana	2	3	5	2*	3	3	5	2	5007	ST5	E5
25	B40908	Uganda	3	4	6	3*	4	2	5	2	5007	ST6	E5
26	B39286	Ghana	4	5	10	4*	10	3	2	2	5007	ST7	E5
27	B39249	Ghana	4	6	6	4*	4	2	2	2	5007	ST8	E5
28	B40014	Tanzania	5	7	7	4*	5	5	3	2	5007	ST9	E5
29	B40406	Tanzania	6	8	7	5*	6	4	2	2	5007	ST10	E5
30	B40408	Tanzania	6	8	7	5*	6	4	2	2	5007	ST10	E5
31	B40391	Tanzania	7	9	6	5*	4	6	2	3	5008	ST11	E5
32	B23976	Kenya	8	10	8	6	7	7	6	4	5160	ST12	E1
33	B32112	Nigeria	8	10	8	6	8	7	6	4	5160	ST13	E1
34	B23983	Kenya	8	10	8	6	8	8	6	4	5160	ST14	E1
35	B24219	Kenya	8	10	8	6	8	8	6	4	5160	ST14	E1
36	B39239	Ghana	8	10	9	6	7	8	6	4	5160	ST15	E1
37	B23987	Kenya	8	10	9	6	8	7	6	4	5160	ST16	E1
38	B24003	Kenya	8	10	9	6	8	7	6	4	5160	ST16	E1
39	B23977	Kenya	8	11	8	6	7	7	6	4	5160	ST17	E1
40	B32314	Nigeria	8	11	8	6	7	8	6	4	5160	ST18	E1
41	B23973	Kenya	8	12	8	6	7	7	6	4	5160	ST19	E1
42	B23982	Kenya	8	12	8	6	7	7	6	4	5160	ST19	E1
43	B40402	Tanzania	8	13	8	6	7	8	6	4	5160	ST20	E1
44	B40006	Tanzania	8	14	8	6	7	7	6	4	5160	ST21	E1
45	B23979	Kenya	9	15	11	7	9	9	7	5	5159	ST22	E2
46	B39302	Ghana	9	15	11	7	9	9	7	5	5159	ST22	E2
47	B39294	Ghana	10	15	10	7	10	9	9	5	5134	ST23	E2
48	B24225	Kenya	11	16	12	8	11	10	8	6	5134	ST24	E2
49	B40910	Uganda	12	17	13	8	12	12	11	6	5133	ST25	E2
50	B40400	Tanzania	12	17	13	8	13	12	10	6	5134	ST26	E2
51	B40396	Tanzania	13	18	14	8	14	11	9	7	5134	ST27	E2
52	B40005	Cameroon	14	19	8	9	15	13	12	8	5139	ST28	E3
53	B24217	Kenya	14	20	15	10	15	13	12	8	5139	ST29	E3
54	B32121	Nigeria	14	20	15	9	15	13	12	8	5139	ST30	E3
55	B37800	Annobón	14	20	15	9	15	13	12	8	5139	ST30	E3
56	B37891	Annobón	14	20	15	9	15	13	12	8	5139	ST30	E3
57	B39289	Ghana	14	20	15	9	15	13	12	8	5139	ST30	E3
58	B24163	Kenya	14	20	15	9	15	14	12	8	5139	ST31	E3
59	B39284	Ghana	14	20	16	10	15	13	12	8	5139	ST32	E3
60	B40398	Tanzania	14	20	17	9	16	13	12	8	5139	ST33	E3
61	B40008	Tanzania	14	21	15	9	15	13	12	8	5139	ST34	E3
62	B32138	Nigeria	14	21	17	10	15	13	12	8	5139	ST35	E3
63	B32120	Nigeria	14	21	17	10	17	13	12	8	5139	ST36	E3
64	B32136	Nigeria	14	21	17	10	17	13	12	8	5139	ST36	E3
65	B37898	Annobón	14	21	17	10	17	13	12	8	5139	ST36	E3
66	B32134	Nigeria	14	21	17	9	16	13	12	8	5139	ST37	E3
67	B39298	Ghana	14	21	17	9	16	13	12	8	5139	ST37	E3
68	B32119	Nigeria	14	1	1	10	17	13	12	8	5139	ST38	E3
69	B23797	Kenya	14	1	1	9	18	13	12	8	5139	ST39	E3
70	B32395	Nigeria	15	22	17	9	18	13	12	8	5139	ST40	E3
71	B39236	Ghana	15	22	17	9	18	13	12	8	5139	ST40	E3
72	B39290	Ghana	15	22	17	9	18	13	12	8	5139	ST40	E3
73	B40011	Tanzania	15	22	17	9	18	13	12	8	5139	ST40	E3
74	B23812	Kenya	16	22	17	9	18	13	12	8	5139	ST41	E3
75	B39300	Ghana	16	21	15	10	17	13	12	8	5139	ST42	E3
76	B39301	Ghana	17	23	18	11	19	15	13	9	5126	ST43	E4
77	B39325	Ghana	18	24	18	11	20	15	13	9	5126	ST44	E4
78	B40407	Tanzania	18	24	18	11	20	15	13	9	5126	ST44	E4
79	B40015	Uganda	18	23	18	11	20	15	13	9	5126	ST45	E4

* *ribC* alleles with a reduced fragment (partially missing).

### Multi-locus sequence typing (MLST)

Eight loci (*ftsZ*, *gltA*, *nuoG*, *ribC*, *rpoB*, *ssrA*, ITS, and 16S rRNA) that have been previously used for bartonella description [27] were selected for MLST characterization of the bartonella isolates. Information on primers is provided in Table 2. A specific fragment of each locus was amplified by PCR for each of the 79 isolates. PCR products of each locus were purified with the QIAquick PCR Purification Kit (Qiagen, Germantown, MD) and sequenced in both directions using an Applied Biosystems Model 3130 Genetic Analyzer (Applied Biosystems, Foster City, CA). Using the Lasergene software package (DNASTAR, Madison, WI), obtained sequences were aligned by each locus and compared between the isolates and with other bat-originated *Bartonella* strains (S1 Table) and known *Bartonella* species (S2 Table). Based on the allelic profile (Table 1), each unique variant was designated as a sequence type (ST) and sequences for the eight loci were concatenated.

**Table 2:** Characteristics of the eight loci evaluated for the straw-colored fruit bat (*Eidolon helvum*)-associated *Bartonella* MLST scheme.

Locus	Forward primer	Reverse primer	Length of analyzed sequence (bp)	No. variable sites	No. alleles
ftsZ	attaatctgcaycggccaga	acvgadacacgaataacacc	885	196	18
gltA	gctatgtctgcattctatca	gatcytcaatcatttctttcca	751	247	24
nuoG	ggcgtgattgttctcgtta	cacgaccacggctatcaat	328	94	18
ribC	taaccgatattggttgtgttgaag	taaagctagaaagtctggcaacataacg	Absence, or 382, or 535	210	11
rpoB	cgcattggcttacttcgtatg	gtagactgattagaacgctg	852	249	20
ssrA	gctatggtaataaatggacaatgaaataa	gcttctgttgccaggtg	282–289	61	15
ITS	cttcagatgatgatcccaagccttctggcg	gaaccgacgaccccctgcttgcaaag a	315–352	210	13
16S rRNA	caggcctaacacatgcaagtc	gggcggwgtgtacaaggc	1172	38	9

### Analysis of nucleotide polymorphism and diversity

Sequence polymorphisms of the eight MLST loci were examined in MEGA v6.06 separately for each *Bartonella* lineage and among all 45 STs. For protein-coding loci (*ftsZ*, *gltA*, *nuoG*, *ribC*, and *rpoB*), the appropriate open reading frame that contained no stop codons was selected for each locus by checking all possible starting positions. To test for evidence of selection in these loci, *d*_N_/*d*_S_ ratios were calculated using the Nei-Gojobori method for each of the loci considering each lineage separately and for all 45 STs together. Nucleotide diversity (*π*), determined by the number of nucleotide differences per site, was calculated as p-distance for all eight loci individually and for concatenated sequences considering each lineage separately and for all 45 STs together. For concatenated sequences, the total length included gap regions and missing genes, thus the pairwise deletion option was used.

### Phylogenetic analysis

A neighbor-joining tree based on the concatenated MLST alleles alone was constructed using the Clustal W program within the Lasergene 11 package of DNASTAR (version 8). An additional phylogeny was constructed, in which known *Bartonella* species and *Bartonella* strains isolated from bats in Old World were included in addition to representative strains from straw-colored fruit bats (*E1*—*E5*, *Ew*), and *Brucella abortus* was included as an outgroup, This phylogeny was inferred using sequences of *ftsZ*, *gltA*, *nuoG*, *ribC*, *rpoB*, *ssrA*, and 16S rRNA; ITS sequences were not included due to the large number of gaps among the strains that could not be resolved. Sequences from each locus were aligned using Clustal X v2.1, trimmed to equal lengths, and concatenated. The best model of nucleotide substitution was determined using MEGA. Based on this model, a maximum-likelihood tree was generated in MEGA with 1000 bootstrap replicates. Due to missing genes among the strains as well as some alignment gaps, we used the pairwise deletion option when inferring the tree.

### Recombination tests

To visualize potential recombination events among the sequence types, a phylogenetic network was inferred from concatenated sequences from the 45 STs using the Neighbor-Net algorithm in SplitsTree v4.13.1 with 1000 bootstrap replicates. Gaps in aligned sequences and from missing genes were included. The pairwise homoplasy index (PHI) was implemented in SplitsTree to test for significant recombination among the isolates.

ClonalFrame v1.1 was used to estimate the relative contribution of recombination and mutation in generating polymorphisms among the 45 *Bartonella* STs. Based on recommendations by Vos and Didelot [32], ITS and genes coding for RNA (*ssrA* and 16S rRNA) were not included because of potential confounding effects of selection on the detection of homologous recombination rates. Therefore, we used only the five protein-coding loci (*ftsZ*, *gltA*, *nuoG*, *ribC*, and *rpoB*) in the study. Two independent runs were performed in ClonalFrame using 200,000 MCMC iterations. The initial mutation rate (*θ*) was set using Watterson's moment estimator while the remaining initial parameters used the default settings in ClonalFrame. We assessed convergence and mixing properties of the dataset through visual inspection of the traces for the likelihood and model parameters.

### Nucleotide sequence accession numbers

Newly-identified alleles from the current study were submitted to GenBank with the following accession numbers: KJ999677 to KJ999694 (*ftsZ*), KM030503 to KM030526 (*gltA*), KM030527 to KM030544 (*nuoG*), KM215178 to KM215188 (*ribC*), KM215189 to KM215208 (*rpoB*), KM233456 to KM233470 (*ssrA*), KM233471 to KM233471 (ITS), and KJ944271 to KJ944279 (16S rRNA).

## Results

### Individual sequence analysis of *gltA* and other loci

All 79 isolates were first compared based on the 751bp *gltA* fragment for the initial identification. The *gltA* sequence alignment revealed 247 variable sites with 24 variants, delineating six unique genogroups with divergence of 7.3–23% among the genogroups and with similarities of ≥ 96.8% within a genogroup. Four genogroups were the same as previously identified in straw-colored fruit bats from Kenya, which were named *E1*, *E2*, *E3*, and *Ew* [18]; whereas the remaining two genogroups were distinct from these four and from any other previously reported *Bartonella* genotypes. Continuing the same naming system proposed for discrimination of *Bartonella* strains discovered in Kenya [18], these two new genogroups were designated as *E4* and *E5*.

Following the initial identification, additional analyses were performed with the other seven loci. Each analyzed locus, except *ribC*, showed that all bartonella isolates obtained from straw-colored fruit bats also fell into the same six genogroups identified by *gltA*. Unexpectedly, analysis of the *ribC* locus revealed a large variation in length of the examined fragment among the isolates. Depending on the group identified by other markers, the examined *ribC* fragment was either fully presented (535bp), or reduced (382bp), or non-amplifiable (PCR negative). The phylogenetic analysis based on *ribC* sequences of the 79 isolates revealed five genogroups related to *E1—E5* clusters that correspond to the grouping by other loci. Absence of the *ribC* locus was indicative for the genogroup *Ew*. All isolates with a reduced *ribC* fragment (partially missing) belonged to the genogroup *E5* (Table 1).

### Allelic profiles, sequence types (ST), and phylogenetic analysis

**Figure 1:**
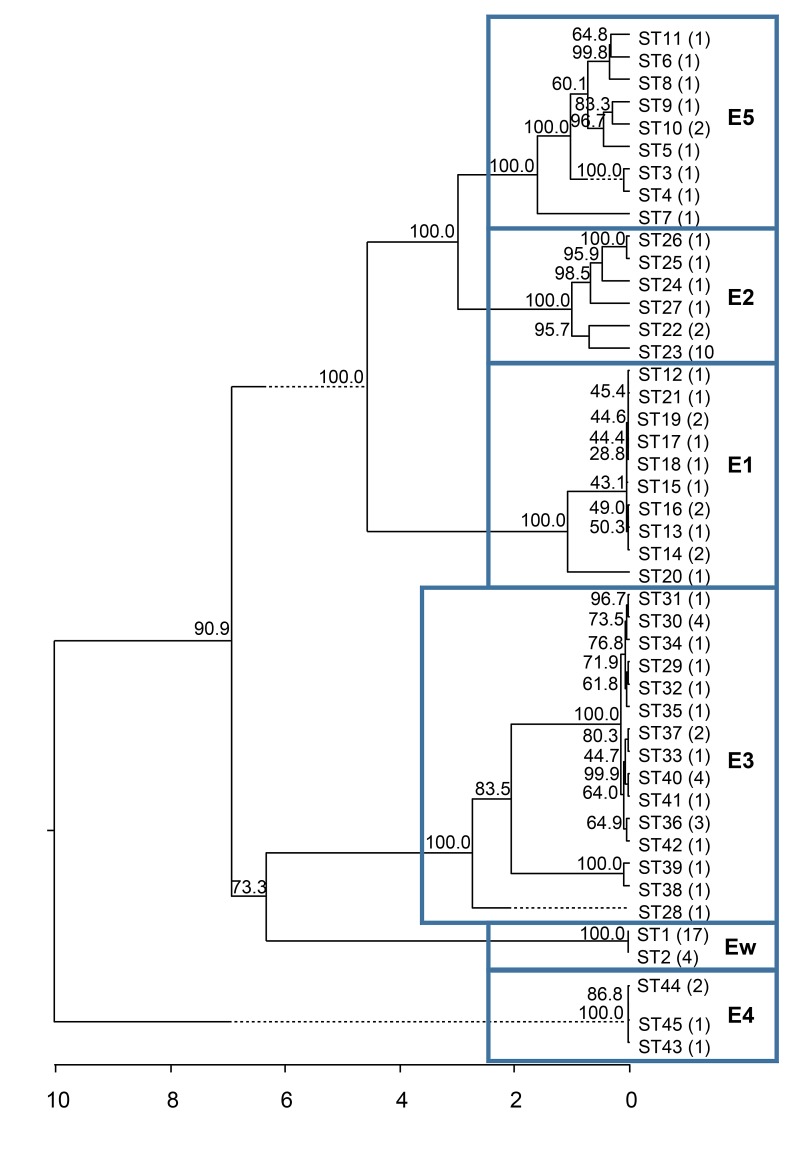
Phylogenetic relationships of the 45 sequence types from 79 bartonella isolates obtained from straw-colored fruit bats (*Eidolon helvum*) in seven African countries/regions. The number of isolates belonging to each sequence type is given in parentheses. The phylogenetic tree was constructed from concatenated sequences (4,622bp—5,160bp) of eight loci (*ftsZ*, *gltA*, *nuoG*, *ribC*, *rpoB*, *ssrA*, ITS, and 16S rRNA) using the neighbor-joining method. Bootstrap values were calculated with 1000 replicates. The sequence types are grouped into six phylogenetic lineages (boxed clades) named as *E1—E5* and *Ew*, with each lineage presumably representing a separate and unique *Bartonella* species.

The size of sequenced fragments ranged between 282bp and 1172bp at different loci. The investigated loci showed different degrees of variation, with 38–249 variable sites and 9–24 alleles (Table 2). The length of concatenated sequences ranged from 4,622bp to 5,160bp as a result of the variation in the fragment length of *ribC* (from 0 to 535bp), ITS (from 315 to 352bp), and *ssrA* (from 282 to 289bp). Based on the allelic profiles, the MLST analysis distinguished 45 sequence types (ST) among the 79 isolates, showing high heterogeneity. Most STs were represented by a single isolate, while some were represented by 2–4 isolates, and ST1 was found in 17 isolates (Table 1). Phylogenetic analysis demonstrated that all of the 45 STs resolved into six lineages, namely, *E1*, *E2*, *E3*, *E4*, *E5*, and *Ew*, to match the names proposed for grouping the strains based on sequences of one locus (Fig. 1). The divergence was 5.1–14% among lineages and ≤ 4.2% within a lineage.

Lineage *Ew* contained 21 isolates of two similar sequence types (ST1 and ST2) with only one nucleotide difference between the two STs. The 17 isolates presenting ST1 were discovered in each of the seven studied countries. ST2 was identified in four isolates from Ghana, Kenya, Nigeria, and Tanzania (one isolate per country) (Table 1). None of the isolates belonging to this lineage possessed the fragment for *ribC*. The lineage *Ew* was very common among the six lineages identified and accounted for 26.6% (21/79) of all analyzed isolates.

Lineage *E1* contained 13 isolates of 10 sequence types (ST12—ST21). The distance among the STs was less than 2.1%. Eight isolates were from Kenya, and the others were from Ghana, Nigeria, and Tanzania (Table 1).

Lineage *E2* included seven isolates of six sequence types (ST22—ST27). The distance among the STs was less than 2.1%. Isolates belonging to this lineage were from Kenya, Ghana, Tanzania, and Uganda (Table 1).

Lineage *E3* contained 24 isolates of 15 sequence types (ST28—ST42). The distance among the STs was less than 4.2%. ST30, ST36, ST37, and ST40 were represented in four, three, two, and three isolates, respectively. The remaining STs were each represented by a single strain.

Lineage *E3* was the most common among all bartonella lineages detected in straw-colored fruit bats, and accounted for 30.4% (26/79) of all isolates analyzed. Notably, mismatches in assignment of isolates to specific ST lineages were observed for a few loci on several occasions. Specifically, isolate B32119 (ST38) from a Kenyan bat and isolate B23797 (ST39) from a Nigerian bat were assigned to lineage *E3* by ST classification, but separate analyses of the *gltA* and *nuoG* sequences of the two isolates indicated their closeness to genogroup *Ew*. Similarly, isolate B40005 (ST28) from a Cameroonian bat was identified as *E3* by ST classification, but was closer to genogroup *E1* by the *gltA* and *nuoG* sequences.

Lineage *E4* contained four isolates of three sequence type (ST43—ST45). These STs are very similar among themselves with distance ≤ 0.1%. The isolates were from Ghana, Tanzania, and Uganda (Table 1).

Lineage *E5* contained 10 isolates of eight sequence types (ST3—ST11). Compared to other lineages, the STs within this lineage were more distant (0.2–3.5%). All isolates/STs in this lineage presented shorter concatenated sequences due to the partial fragment missing in *ribC*. One isolate (B39286) from this lineage also showed a mismatch in lineage assignment when analyzed by individual loci. The isolate was from a Ghanaian bat and belonged to lineage *E5* by ST classification, but assigned to genogroup *E2* by *rpoB* and *nuoG* sequences.

**Figure 2:**
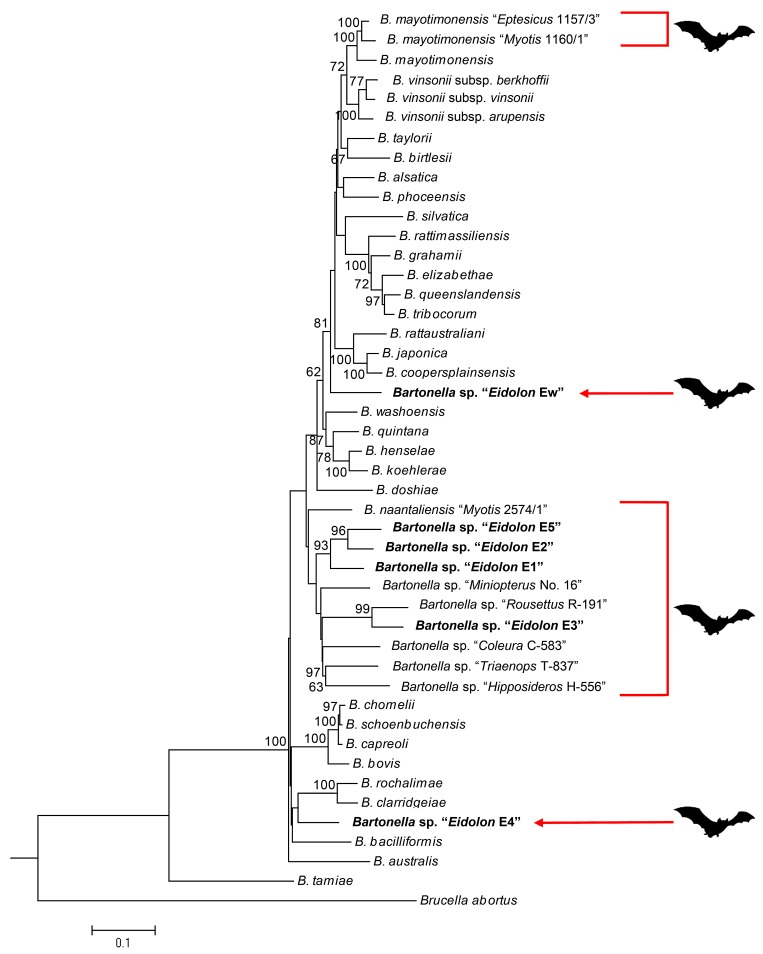
Phylogenetic relationship of straw-colored fruit bats (*E*. *helvum*) with known *Bartonella* species and other bat-associated *Bartonella* strains. The maximum-likelihood tree was inferred using concatenated sequence of seven loci (*ftsZ*, *gltA*, *nuoG*, *ribC*, *rpoB*, *ssrA*, and 16S rRNA) from 31 *Bartonella* species, other bat-associated *Bartonella* strains, and 6 sequence types from *E*. *helvum* representing the lineages *E1*—*E5* and *Ew* (bold text). Groups of bat-associated *Bartonella* strains are indicated with a bat silhouette. The numbers at the nodes correspond to bootstrap values greater than 60% based on 1000 replicates.

Based on MEGA results, the best nucleotide substitution model for the concatenated sequences of *gltA*, *ftsZ*, *nuoG*, *ribC*, *rpoB*, *ssrA*, and 16S rRNA was determined to be GTR+G+I [33]. Comparison with known *Bartonella* species and other bat-associated *Bartonella* strains demonstrated that lineages *E1*, *E2*, *E3*, and *E5* belong to a clade that includes other *Bartonella* strains found in Old World bats, while *E4* and *Ew* appear to have arisen independently (Fig. 2).

### Patterns of selection and diversity in nucleotide sequences

The *d*_N_/*d*_S_ ratios calculated for protein-coding sequences ranged from 0.022 for *ftsZ* to 0.242 for *ribC* when comparing all 45 STs. The values for each locus differed when each *Bartonella* lineage was analyzed separately. Nucleotide diversity among STs varied by locus, ranging from 1.02% in 16S rRNA to 14.38% in *ribC*. Among the *Bartonella* lineages, *E2* and *E5* had the highest nucleotide diversity when all loci were considered together, with 1.56% and 1.24%, respectively, while *Ew* had the lowest (0.02%).

### Recombination test

**Figure 3:**
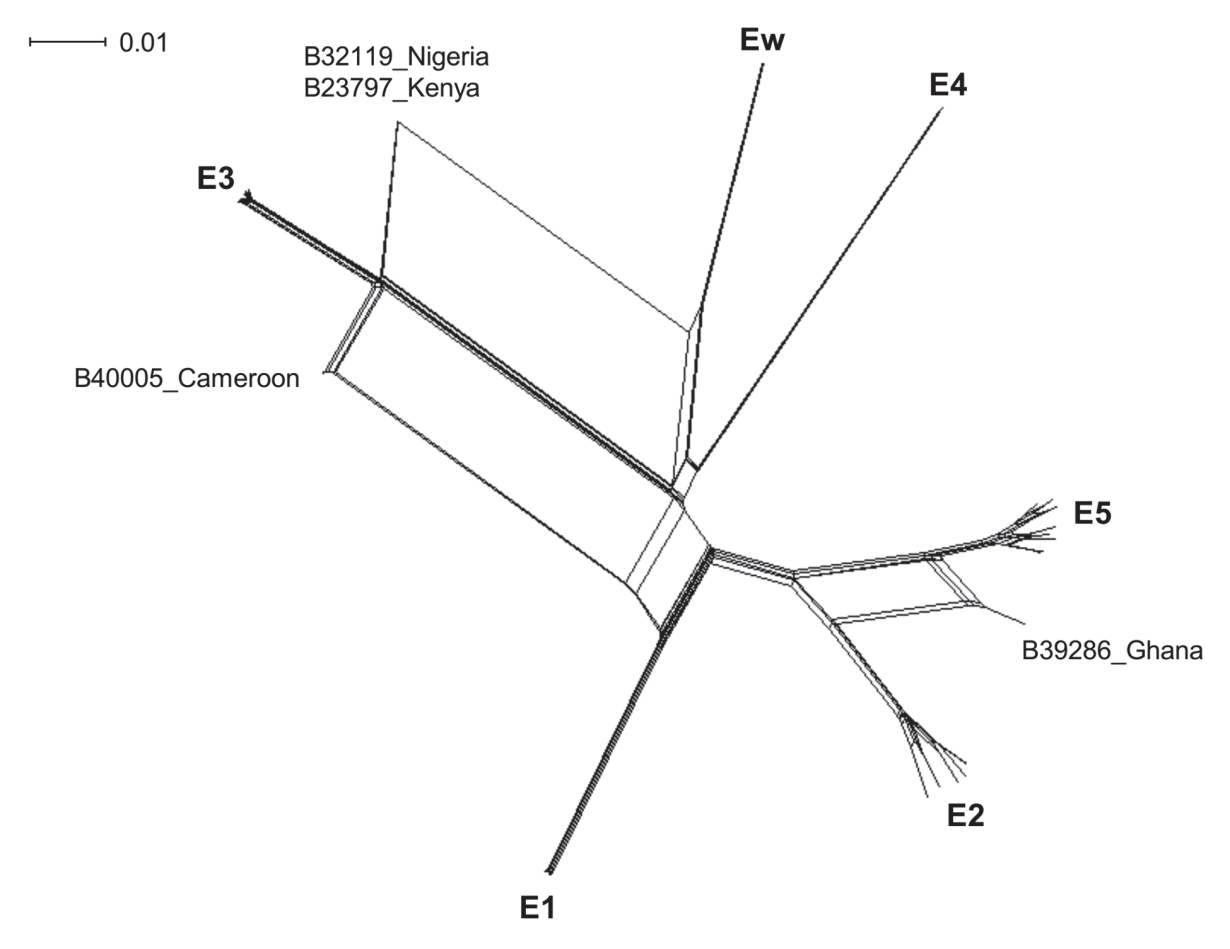
Network phylogeny of the 45 bartonella sequence types obtained from *Eidolon helvum*. The network was constructed in SplitsTree using the NeighborNet algorithm based on concatenated sequences of eight loci (*ftsZ*, *gltA*, *nuoG*, *ribC*, *rpoB*, *ssrA*, ITS, and 16S rRNA). Clusters of sequence types were named according to phylogenetic lineages (*E1*—*E5*, *Ew*). Individual isolate labels indicate samples with mixed ancestry due to possible recombination.

A network phylogeny based on the concatenated sequences for the 45 STs in SplitsTree (v4.13.1) shows that the 45 STs fall into the same six identified lineages identified by single loci. However, isolates B32119 from Nigeria, B23797 from Kenya, B40005 from Cameroon, and B39286 from Ghana are split from the network, reflecting their mixed ancestry (Fig. 3). The pairwise homoplasy index (PHI) [34] found significant evidence of recombination in the network (mean = 0.2, variance = 4.5x10^–6^, p < 0.01). Using ClonalFrame, the *r/m* value (the ratio of probabilities that a site is altered by recombination or mutation) was estimated to be 1.14 (95% CI 0.63, 1.82) and 1.06 (95% CI 0.53, 1.67) based on two independent runs (average = 1.1), while the *ρ*/*θ* value (the ratio of rates at which recombination and mutation occur) was estimated to be 0.049 (95% CI 0.023, 0.087) and 0.037 (95% CI 0.016, 0.065).

## Discussion

Straw-colored fruit bats have been reported to be commonly infected with multiple *Bartonella* strains based on *gltA* sequences [18,22]. In this study, we developed a MLST scheme using eight loci which have been commonly used for *Bartonella* species description to characterize 79 bartonella isolates obtained from straw-colored fruit bats that were captured in different regions across Africa to better elucidate the genetic diversity among these isolates.

The analyses of selection pattern showed none of the *d*_N_/*d*_S_ ratios exceeded 1. This suggested that all of the protein-coding loci in the study are undergoing purifying selection. The analyzed bartonella isolates exhibited a high level of heterogeneity with 9–24 alleles identified by different loci. The MLST scheme resolved 45 STs among the 79 isolates and these isolates/STs clustered into six distinct lineages (*E1—E5*, and *Ew*) with genetic distances of 5.1–14% among the lineages. MLST has been regarded as a highly discriminatory tool by a number of previous studies. According to the widely accepted criteria for a new bacterial species based on nucleotide divergence greater than 5% in housekeeping genes [31], the MLST analyses in the present study demonstrated that each lineage identified in straw-colored fruit bats in this study likely represents a new *Bartonella* species. These *Bartonella* species have never been found in other bat species, suggesting their specific co-adaptation to *Eidolon* bats, though future studies are necessary to confirm this. The performed analyses allow us to conclude that there are at least six *Bartonella* species associated with straw-colored fruit bats. Phylogenetic analysis based on multiple loci demonstrates that four of these lineages, *E1*, *E2*, *E3*, and *E5*, belong to a phylogenetic lineage that includes other *Bartonella* strains isolated from Old World bats [18,21–23], while *E4* and *Ew* appear to have evolved independently.

Lineages *E3* and *Ew*, containing 24 and 21 isolates, respectively, and accounting for 57% of all isolates, are likely to be the more common *Bartonella* species circulating among this bat species. However, because the original bat samples from which the analyzed bartonella isolates obtained were collected in different settings and during different seasons by multiple investigators, there could be some bias in temporal or seasonal variations. Furthermore, the culturing procedure that was necessary for performing such bacterial classification may also contribute to misrepresentation of comparative prevalence of *Bartonella* species as some of them could hardly be cultured [35]. Due to the limited number of isolates selected from each part of Africa, we did not attempt to investigate any patterns in the geographic distribution of *Bartonella* species in this study. The presented classification, however, can be used for future studies aimed at describing geographic patterns of bartonella distribution among *Eidolon* bats.

Most lineages consisted of multiple STs (from 4 to 14). Nevertheless, lineage *Ew* consisted of only two STs with a large number of isolates, and in fact there is only one nucleotide difference between the two STs. This indicates a lower diversity of allelic polymorphisms in the isolates from this lineage and allows a speculation that *Ew* has diverged more recently compared to the other *Bartonella* lineages. Further, our observations of partially missing or non-amplifiable sequences of *ribC* suggests gene loss, a common evolutionary process in bacteria [36], possibly has occurred and resulted in the appearance of two new *Bartonella* species (*E5* and *Ew*). As a housekeeping gene, *ribC* participates in the regulation of riboflavin biosynthesis. The non-amplifiability of the gene might not be true but suggest the gene is in some undetected form. If this gene is truly missing, its loss may be compensated by the fact that bartonellae are intracellular parasites which can perhaps utilize host riboflavin or they have lost the need of it. If this were true, fitness costs may lead to selection against this superfluous genetic material [37]. Overall, little is known about the mechanisms of regulation of bacterial riboflavin genes [38]. Although we cannot explain biological consequences of the loss of *ribC*, the 'presence/absence' characteristics make *ribC* a marker for the differentiation and classification of *Eidolon*-associated *Bartonella* strains. In addition, the dramatic variation in *ribC* among the straw-colored fruit bat-associated *Bartonella* species presents an interesting model for understanding evolutionary trends in developing host-bacterial parasite relations [36].

Another intriguing observation in this study was the set of mismatches between MLST lineages and some genogroups comparing phylograms built based either on entire concatenated sequences or individual locus sequences for four isolates. Particularly, three isolates belonging to *E3* based on the ST classification were either *Ew* (two isolates) or *E1* (one isolate) by *nuoG* or *gltA* alone and one isolate of *E5* by ST classification would be *E2* by *nuoG* or *rpoB* alone. The loci *rpoB* and *gltA* have been shown to be the most potent for species discrimination in other studies [31]. According to our observations in the present study, an analysis limited to two loci may still lead to a misclassification. Thus characterization of multiple loci should be preferably used for *Bartonella* species identification. The mismatches between lineages and genogroups identified by different loci indicate that recombination and/or lateral gene transfer events between different *Bartonella* species are ongoing processes.

Our recombination test results indicate that while recombination happens infrequently, an individual recombination event introduces a large number of polymorphisms. Recombination in *rpoB*, *gltA*, and other housekeeping loci has been observed in other *Bartonella* species [29,30,39]. Estimated *r/m* values for *B*. *grahamii* alone (1.7 [38] and 6.81 [30]), *B*. *taylorii* alone (3.77 [27]), or *B*. *grahamii* and *B*. *taylorii* considered together (4.06 [30]) compare favorably with our estimate for recombination among six species of *Bartonella* in *E*. *helvum* (*r/m* = 1.1). These estimates contrast with results from *B*. *henselae* (*r/m* = 0.1) [40] and the perception that intracellular bacteria have low rates of recombination and horizontal gene transfer [32]. Intermediate to high rates of recombination in these studies suggest that multiple *Bartonella* species may coexist at some point in the infection cycle, potentially in their mammalian hosts and/or arthropod vectors, thus facilitating gene exchange. Moreover, gene exchange appears to play an important role in generating sequence diversity among co-circulating *Bartonella* species. Further studies that could measure co-infection of *Bartonella* species and resulting rates of recombination would help to explain these interesting dynamics.

Our study provides information on the genetic diversity of straw-colored fruit bat-associated *Bartonella* species that can be used for the discrimination of the lineages at the level corresponding to separate species. Additional information is needed to understand the interaction between these *Bartonella* species and their bat hosts under natural conditions, as well as their putative ectoparasite vectors [41]. A biological approach to define *Bartonella* species has been discussed [42] with emphasis on host-association as a phenomenon that promotes biological isolation of *Bartonella* species. However, such an approach has limitations for situations when several *Bartonella* species are associated with one animal host. A similar situation was also described among *Bartonella* strains in grasshopper mice [43] and cotton rats [44]. Nevertheless, among all mammalian species described as a host of one or multiple *Bartonella* species, the number of presumptive species associated with *E*. *helvum* bats is clearly the highest. Although there is no evidence suggesting that *Bartonella* species associated with African fruit bats may cause illnesses in humans or in bats themselves, a recent study reported the presence of Bartonella mayotimonensis, a reported etiologic agent of endocarditis in humans, in the Daubenton's bat (*Myotis daubentonii*) and the Northern bat (Eptesicus nilssonii) in Europe [23], suggesting a potential role of bats as reservoirs for human bacterial pathogens. It is important to understand the role of straw-colored fruit bats in biotic communities and their importance as reservoir hosts contributing to the maintenance and transmission of bartonellae to other animals and humans.

The great diversity of *Bartonella* species associated with *E*. *helvum*, however, clearly presents interesting questions from the perspective of microbial evolution. What are the mechanisms that generate and maintain such diversity? How can these bacteria share the same ecological niche? Analyzing such relationships, Chan and Kosoy [45] hypothesized that co-circulation of independent *Bartonella* species in populations of one host species may represent an escape mechanism to circumvent the host immune responses. We ultimately believe this system will be very informative for understanding the evolution of bacteria in a host-vector-pathogen system.

## Supporting Information

S1 Table GenBank accession numbers for *ftsZ*, *gltA*, *nuoG*, *ribC*, *rpoB*, *ssrA*, ITS, and 16S rRNA sequences for bat-associated *Bartonella* strains. (DOCX)

S2 Table GenBank accession numbers for *ftsZ*, *gltA*, *nuoG*, *ribC*, *rpoB*, *ssrA*, ITS, and 16S rRNA sequences of reference *Bartonella* species. (DOCX)

## References

[1] Welch DF, Pickett DA, Slater LN, Steigerwalt AG, Brenner DJ (1992) *Rochalimaea henselae* sp. nov., a cause of septicemia, bacillary angiomatosis, and parenchymal bacillary peliosis. J Clin Microbiol 30: 275–280. 1537892 10.1128/jcm.30.2.275-280.1992 PMC265045

[2] Daly JS, Worthington MG, Brenner DJ, Moss CW, Hollis DG, et al (1993) *Rochalimaea elizabethae* sp. nov. isolated from a patient with endocarditis. J Clin Microbiol 31: 872–881. 7681847 10.1128/jcm.31.4.872-881.1993 PMC263580

[3] Bass JW, Vincent JM, Person DA (1997) The expanding spectrum of bartonella infections II. Cat scratch disease. Pediatr Infect Dis J 16: 163–179. 9041596 10.1097/00006454-199702000-00002

[4] Kerkhoff FT, Bergmans AM, van Der Zee A, Rothova A (1999) Demonstration of *Bartonella grahamii* DNA in ocular fluids of patient with neuroretinitis. J Clin Microbiol 37: 4034–4038. 10565926 10.1128/jcm.37.12.4034-4038.1999 PMC85873

[5] Roux V, Eykyn SJ, Wyllie S, Raoult D (2000) *Bartonella vinsonii* subsp. *berkhoffii* as an agent of a febrile blood culture-negative endocarditis in a human. J Clin Microbiol 38: 1698–1700. 10747175 10.1128/jcm.38.4.1698-1700.2000 PMC86533

[6] Kosoy MY, Murray M, Gilmore RD, Bai Y, Gage K (2003) Bartonella strains from ground squirrels are identical to *Bartonella washoensis* isolated from a human patient. J Clin Microbiol 41: 645–650. 12574261 10.1128/JCM.41.2.645-650.2003 PMC149662

[7] Eremeeva ME, Gerns HL, Lydy SL, Goo JS, Ryan ET, et al (2007) Bacteremia, fever, and splenomegaly caused by a newly recognized Bartonella species. N Engl J Med 256: 2381–2387. 10.1056/NEJMoa065987 17554119

[8] Kosoy M, Morway C, Sheff K, Bai Y, Colborn J, et al (2008) *Bartonella tamiae* sp. nov., a newly recognized pathogen isolated from human patients from Thailand. J Clin Microbiol 46: 772–775. 18077632 10.1128/JCM.02120-07 PMC2238074

[9] Welch DF, Carroll KC, Hofmeister EK, Persing DH, Robison DA, et al (1999) Isolation of a new subspecies, *Bartonella vinsonii* subsp. *arupensis*, from a cattle rancher: identity with isolates found in conjunction with *Borrelia burgdorferi* and *Babesia microti* among naturally infected mice. J Clin Microbiol 37: 2598–2601. 10405408 10.1128/jcm.37.8.2598-2601.1999 PMC85292

[10] Garcia-Caceres U, Garcia FU (1991) Bartonellosis: an immunodepressive disease and the life of Daniel Alcides Carrion. Am J Clin Pathol 95: s58–s66. 2008885

[11] Chomel BB, Kasten RW, Floyd-Hawkins KA, Chi B, Yamamoto K, et al (1996). Experimental transmission of *Bartonella henselae* by the cat flea. J Clin Microbiol 34: 1952–1956. 8818889 10.1128/jcm.34.8.1952-1956.1996 PMC229161

[12] Higgins JA, Radulovic S, Jaworski DC, Azad AF (1996) Acquisition of the cat scratch disease agent *Bartonella henselae* by cat fleas (Siphonaptera: Pulicidae). J Med Entomol 33: 490–495. 8667399 10.1093/jmedent/33.3.490

[13] Pappalardo BL, Correa MT, York CC, Peat CY, Breitschwerdt EB (1997) Epidemiologic evaluation of the risk factors associated with exposure and seroreactivity to *Bartonella vinsonii* in dogs. Am J Vet Res 58: 467–471. 9140552

[14] Roux V, Raoult D (1999) Body lice as tools for diagnosis and surveillance of reemerging diseases. J Clin Microbiol 37: 596–599. 9986818 10.1128/jcm.37.3.596-599.1999 PMC84482

[15] Billeter SA, Sangmaneedet S, Kosakewich RC, Kosoy MY (2012) *Bartonella* species in dogs and their ectoparasites from Khon Kaen Province, Thailand. Southeast Asian J Trop Med Public Health 43: 1186–1192. 23431825

[16] Luis AD, Hayman DTS, O’Shea TJ, Cryan PM, Gilbert AT, et al (2013) A comparison of bats and rodents as reservoirs of zoonotic viruses: are bats special? Proc Biol Sci 280: 20122753 10.1098/rspb.2012.2753 23378666 PMC3574368

[17] Concannon R, Wynn-Owen K, Simpson VR, Birtles RJ (2005) Molecular characterization of haemoparasites infecting bats (Microchiroptera) in Cornwall, UK. Parasitology 131: 489–496. 16174413 10.1017/S0031182005008097

[18] Kosoy M, Bai Y, Lynch T, Kuzmin I, Niezgoda M, et al (2010) *Bartonella* spp. in bats, Kenya. Emerg Infect Dis 16: 1875–1881. 10.3201/eid1612.100601 21122216 PMC3294596

[19] Bai Y, Kosoy M, Recuenco S, Alvarez D, Moran D, et al (2011) *Bartonella* spp. in bats, Guatemala. Emerg Infects Dis 17: 1269–1271. 10.3201/eid1707.101867 21762584 PMC3381397

[20] Bai Y, Recuenco S, Turmelle A, Osikowicz LM, Gomez J, et al (2012) Prevalence and diversity of *Bartonella* spp. in bats in Peru. Am J Trop Med & Hyg 87: 518–523. 22826480 10.4269/ajtmh.2012.12-0097 PMC3435358

[21] Lin JW, Hsu YM, Chomel BB, Lin LK, Pei JC, et al (2012) Identification of novel *Bartonella* spp. in bats and evidence of Asian gray shrew as a new potential reservoir of *Bartonella* . Vet Microbiol 156: 119–126. 10.1016/j.vetmic.2011.09.031 22005177 PMC7126237

[22] Kamani J, Baneth G, Mitchell M, Mumcuoglu KY, Gutiérrez R, et al (2014) *Bartonella* species in bats (Chiroptera) and bat flies (Nycteribiidae) from Nigeria, West Africa. VBZD 14: 625–632. 10.1089/vbz.2013.1541 PMC4170809 25229701

[23] Veikkolainen V, Vesterinen EJ, Lilley TM, Pulliainen AT (2014) Bats as reservoir hosts of human bacterial pathogen, *Bartonella mayotimonensis* . Emerg Infect Dis 20: 960–967. 10.3201/eid2006.130956 24856523 PMC4036794

[24] Hayman DTS, McCrea R, Suu-Ire R, Wood JLN, Cunningham AA, et al (2012) Straw-colored fruit bat demography in Ghana. J Mammal 93: 1393–1404 23525358 10.1644/11-MAMM-A-270.1 PMC3605799

[25] Arvand M, Raoult D, Feil EJ (2010) Multi-locus sequence typing of a geographically and temporally diverse sample of the highly clonal human pathogen *Bartonella quintana* . PLoS One 5: e9765 10.1371/journal.pone.0009765 20333257 PMC2841634

[26] Chaloner GL, Ventosilla P, Birtles RJ (2011) Multi-locus sequence analysis reveals profound genetic diversity among isolates of the human pathogen *Bartonella bacilliformis* . PLoS Negl Trop Dis 7: e1248 10.1371/journal.pntd.0001248 21811647 PMC3139668

[27] Mietze A, Morick D, Köhler H, Harrus S, Dehio C, et al (2011) Combined MLST and AFLP typing of *Bartonella henselae* isolated from cats reveals new sequence types and suggests clonal evolution. Vet Microbiol 148: 238–245. 10.1016/j.vetmic.2010.08.012 20863631

[28] Bai Y, Malania L, Castillo DA, Moran D, Boonmar S, et al (2013) Global distribution of bartonella infections in domestic bovine and characterization of *Bartonella bovis* strains using multi-locus sequence typing. PloS One. 10.1371/journal.pone.0080894 PMC3836770 24278342

[29] Paziewska A, Harris PD, Zwolińska L, Bajer A, Siński E (2011) Recombination within and between species of the alpha proteobacterium *Bartonella* infecting rodents. Microb Ecol 61: 134–145. 10.1007/s00248-010-9735-1 20740281 PMC3011088

[30] Buffet JP, Pisanu B, Brisse S, Roussel S, Félix B, et al (2013) Deciphering *Bartonella* diversity, recombination, and host specificity in a rodent community. PLoS One 8: e68956 10.1371/journal.pone.0068956 23894381 PMC3722228

[31] La Scola B, Zeaiter Z, Khamis A, Raoult D (2003) Gene-sequence-based criteria for species definition in bacteriology: the Bartonella paradigm. Trends Microbiol 11: 318–321. 12875815 10.1016/s0966-842x(03)00143-4

[32] Vos M, Didelot X (2009) A comparison of homologous recombination rates in bacteria and archaea. ISME J 3: 199–208. 10.1038/ismej.2008.93 18830278

[33] Tavaré S (1986) Some probabilistic and statistical problems in the analysis of DNA sequences. Lectures Math Life Sci 17: 57–86.

[34] Bruen TC, Phillippe H, Bryant D (2006) A simple and robust statistical test for detecting the presence of recombination. Genetics 172: 2665–2681. 16489234 10.1534/genetics.105.048975 PMC1456386

[35] Gundi VA, Billeter SA, Rood MP, Kosoy MY (2012) *Bartonella* spp. in rats and zoonoses, Los Angeles, California, USA. Emerg Infect Dis 18: 631–633. 10.3201/eid1804.110816 22469313 PMC3309692

[36] Zhu Q, Kosoy M, Olival KJ, Dittmar K (2014) Horizontal Transfers and Gene Losses in the Phospholipid Pathway of *Bartonella* Reveal Clues about Early Ecological Niches. Genome Biol Evol 6: 2156–2169. 10.1093/gbe/evu169 25106622 PMC4159011

[37] Koskiniemi S, Sun S, Berg OG, Andersson DI (2012) Selection-driven gene loss in bacteria. PLoS Genet 8: e1002787 10.1371/journal.pgen.1002787 22761588 PMC3386194

[38] Vitreschak AG, Rodionov DA, Mironov AA, Gelfand MS (2002) Regulation of riboflavin biosynthesis and transport genes in bacteria by transcriptional and translational attenuation. Nucleic Acids Res. 30: 3141–351. 12136096 10.1093/nar/gkf433 PMC135753

[39] Berglund EC, Ellegaard K, Granberg F, Xie Z, Maruyama S, et al (2010) Rapid diversification by recombination in *Bartonella grahamii* from wild rodents in Asia contrasts with low levels of genomic divergence in Northern Europe and America. Mol Ecol 19: 2241–2255. 10.1111/j.1365-294X.2010.04646.x 20465583

[40] Arvand M, Feil EJ, Giladi M, Boulouis HJ, Viezens J (2007) Multi-locus sequence typing of *Bartonella henselae* isolates from three continents reveals hypervirulent and feline-associated clones. PLoS One 2: e1346 18094753 10.1371/journal.pone.0001346 PMC2147075

[41] Billeter SA, Hayman DTS, Peel A, Baker KS, Cunningham AA, et al (2012) *Bartonella* species in bat flies (Diptera: Nycteribiidae) from western Africa. Parasitology 139: 324–329. 10.1017/S0031182011002113 22309510

[42] Kosoy M, Hayman DT, Chan KS (2012) Bartonella bacteria in nature: where does population variability end and a species start? Infect Genet Evol 12: 894–904. 10.1016/j.meegid.2012.03.005 22449771

[43] Bai Y, Kosoy MY, Cully JF, Bala T, Ray C, et al (2007) Acquisition of nonspecific *Bartonella* strains by the northern grasshopper mouse (*Onychomys leucogaster*). FEMS Microbiol Ecol 61: 438–48. 17672850 10.1111/j.1574-6941.2007.00364.x

[44] Kosoy M, Mandel E, Green D, Marston E, Jones D, et al (2004) Prospective studies of *Bartonella* of rodents. Part II. Diverse infections in a single rodent community. Vector Borne Zoonotic Dis 4: 296–305. 15671736 10.1089/vbz.2004.4.296

[45] Chan KS, Kosoy M (2010) Analysis of multi-strain *Bartonella* pathogens in natural host population—do they behave as species or minor genetic variants? Epidemics 2: 165–172. 10.1016/j.epidem.2010.08.002 21352787

