# Supplementary material for: Classification of Bartonella Strains Associated with Straw-Colored Fruit Bats (Eidolon helvum) across Africa Using a Multi-locus Sequence Typing Platform
Source: PLoS Negl Trop Dis. 2015 Jan 30;9(1):e0003478. doi: 10.1371/journal.pntd.0003478 (PMC4311972; doi:10.1371/journal.pntd.0003478)
Supplement: S1 Table — (DOCX) [file pntd.0003478.s001.docx]

| **Table S1.** GenBank accession numbers for *ftsZ*, *gltA*, *nuoG*, *ribC*, *rpoB*, *ssrA*, ITS, and 16S rRNA sequences for bat-associated *Bartonella* strains. | | | | | | | | | | | |
| --- | --- | --- | --- | --- | --- | --- | --- | --- | --- | --- | --- |
|  |  |  |  |  |  |  |  |  |  |  |  |
|  |  |  |  | **Accession numbers** | | | | | | | |
| **Species** | **Strain** | **Country** | **Host** | ***ftsZ*** | ***gltA*** | ***nuoG*** | ***ribC*** | ***rpoB*** | ***ssrA*** | **ITS** | **16S rRNA** |
| *Bartonella* sp. "*Rousettus* R-191" | R-191 | Kenya | *Rousettus aegyptiacus* | HM363769 | HM363764 | KM387321 | HM363779 | HM363774 | KM382247 | KM382255 | HM363784 |
| *Bartonella* sp. "*Coleura* C-583" | C-583 | Kenya | *Coleura afra* | HQ832883 | HM545136 | - | - | - | KM382248 | KM382256 | - |
| *Bartonella* sp. "*Triaenops* T-837" | T-837 | Kenya | *Triaenops persicus* | KM382253 | HM545138 | KM382251 | - | - | KM382249 | KM382257 | - |
| *Bartonella* sp. "*Hipposideros* H-556" | H-556 | Kenya | *Hipposideros* sp. | KM382254 | HM545137 | KM382252 | - | - | KM382250 | KM382258 | - |
| *Bartonella* sp. "*Miniopterus* No. 16 " | No. 16 | Taiwan | *Miniopterus schreibersii* | JF500506 | JF500522 | - | JF500538 | JF500554 | - | - | JF500560 |
| *B. mayotimonensis* "*Eptesicus* 1157/3" | 1157/3 | Finland | *Eptesicus nilssoni* | KF003121 | KF003115 | - | - | KF003118 | KF003119 | KF003117 | KF003116 |
| *B. mayotimonensis* "*Myotis* 1160/1" | 1160/1 | Finland | *Myotis daubentonii* | KF003128 | KF003122 | - | - | KF003125 | KF003126 | KF003124 | KF003123 |
| *B. naantaliensis* "*Myotis* 2574/1" | 2574/1 | Finland | *Myotis daubentonii* | KF003135 | KF003129 | - | - | KF003132 | KF003133 | KF003131 | KF003130 |
