# Supplementary material for: Classification of Bartonella Strains Associated with Straw-Colored Fruit Bats (Eidolon helvum) across Africa Using a Multi-locus Sequence Typing Platform
Source: PLoS Negl Trop Dis. 2015 Jan 30;9(1):e0003478. doi: 10.1371/journal.pntd.0003478 (PMC4311972; doi:10.1371/journal.pntd.0003478)
Supplement: S2 Table — (DOCX) [file pntd.0003478.s002.docx]

| **Table S2.** GenBank accession numbers for *ftsZ*, *gltA*, *nuoG*, *ribC*, *rpoB*, *ssrA*, ITS, and 16S rRNA sequences of reference *Bartonella* species. | | | | | | | | | |
| --- | --- | --- | --- | --- | --- | --- | --- | --- | --- |
|  |  | **Accession numbers** | | | | | | | |
| **Species** | **Strain** | ***ftsZ*** | ***gltA*** | ***nuoG*** | ***ribC*** | ***rpoB*** | ***ssrA*** | **ITS** | **16S rRNA** |
| *B. alsatica* | IBS 382 | AF467763 | AF204273 | EF659935 | AY116630 | AF165987 | JN029776 | AF312506 | AJ002139 |
| *B. australis* | AUST/NH1 | CP003123 | CP003123 | CP003123 | CP003123 | CP003123 | - | DQ538396 | NR_115816 |
| *B. bacilliformis* | KC583 | NC_008783 | NC_008783 | NC_008783 | NC_008783 | NC_008783 | NC_008783 | L26364 | NC_008783 |
| *B. birtlesii* | IBS 325 | AM690313 | AF204272 | - | AM690314 | AB196425 | JN029775 | AY116640 | AF204274 |
| *B. bovis* | 91-4 | AF467761 | AF293394 | EF659938 | AY116637 | AY166581 | JN029767 | - | NR_025121 |
| *B. capreoli* | IBS 193 | AB290192 | AF293392 | - | AB290194 | AB290188 | - | EU098130 | NR_025120 |
| *B. chomelii* | A828 | AB290193 | AY254308 | - | AB290195 | AB290189 | JN029773 | AB498010 | NR_025736 |
| *B. clarridgeiae* | 73 | NC_014932 | NC_014932 | NC_014932 | NC_014932 | NC_014932 | - | - | NC_014932 |
| *B. coopersplainsensis* | AUST/NH20 | EU111781 | EU111803 | - | - | EU111792 | - | EU111770 | NR_116177 |
| *B. doshiae* | R18 | AF467754 | AF207827 | - | AY116627 | AF165991 | JN029768 | AJ269786 | Z31351 |
| *B. elizabethae* | F9251 | AF467760 | Z70009 | EF659940 | AY116633 | AF165992 | JN029774 | L35103 | L01260 |
| *B. grahamii* | as4aup | NC_012846 | NC_012846 | NC_012846 | NC_012846 | NC_012846 | NC_012846 | - | NC_012846 |
| *B. henselae* | Houston-1 | NC_005956 | NC_005956 | NC_005956 | NC_005956 | NC_005956 | JN029785 | L35101 | NC_005956 |
| *B. japonica* | Fuji 18-1 | AB440633 | AB242289 | - | AB440635 | AB242288 | JN029784 | AB498007 | NR_112790 |
| *B. koehlerae* | C-29 | AF467755 | AF176091 | EF659942 | AY116634 | AY166580 | JN029769 | - | AF076237 |
| *B. mayotimonensis* | EYL-2008 | FJ376734 | FJ376732 | - | - | FJ376736 | - | FJ376735 | FJ376733 |
| *B. phoceensis* | 16120 | AY515135 | AY515126 | - | AY515138 | AY515132 | JN029770 | AY515123 | NR_115254 |
| *B. queenslandensis* | AUST/NH12 | EU111776 | EU111798 | - | - | EU111787 | - | EU111765 | NR_116176 |
| *B. quintana* | Toulouse | NC_005955 | NC_005955 | NC_005955 | NC_005955 | NC_005955 | - | L35100 | NC_005955 |
| *B. rattaustraliani* | AUST/NH14 | EU111774 | EU111796 | - | - | EU111785 | - | EU111763 | EU111752 |
| *B. rattimassiliensis* | 15908 | AY515133 | AY515124 | - | AY515136 | AY515130 | - | AY515121 | NR_115255 |
| *B. rochalimae* | ATCC BAA-1498 | FN645461 | FN645459 | - | FN645459 | FN645459 | - | DQ683199 | NR_115858 |
| *B. schoenbuchensis* | R1 | AF467765 | AJ278183 | FN645509 | AY116628 | AY167409 | JN029772 | - | AJ278187 |
| *B. silvatica* | Fuji 23-1 | AB440637 | AB242287 | - | AB440639 | AB242292 | JN029782 | AB498008 | NR_112791 |
| *B. tamiae* | Th239 | DQ395178 | DQ395177 | - | - | EF091855 | JN029780 | DQ395180 | DQ395176 |
| *B. taylorii* | M6 | AF467756 | Z70013 | EF659943 | AY116635 | AF165995 | JN029781 | AJ269784 | Z31350 |
| *B. tribocorum* | CIP 105476 | NC_010161 | NC_010161 | NC_010161 | NC_010161 | NC_010161 | - | AF312505 | NC_010161 |
| *B. vinsonii* subsp. *arupensis* | OK94-513 | AF467758 | AF214557 | EF659936 | AY116631 | AY166582 | JN029783 | AF312504 | AF214558 |
| *B. vinsonii* subsp. *berkhoffii* | 93-CO1 | AF467764 | BVU28075 | EF659937 | AF548031 | AF165989 | JN394654 | AF167988 | U26258 |
| *B. vinsonii* subsp. *vinsonii* | Baker | AF467757 | Z70015 | EF659944 | AY116636 | AF165997 | JN029777 | L35102 | M73230 |
| *B. washoensis* | Sb944nv | AB292598 | AF470616 | EF659945 | AB292599 | AB292596 | JN029786 | AB674253 | AB292597 |
| *Brucella abortus* | 9-941 | AE017223 | AE017223 | AE017223 | AE017223 | AE017223 | - | - | NR_102910 |
